# Comfort-Promoting Interventions for the Elderly in Hospital Settings

**DOI:** 10.3390/geriatrics9060157

**Published:** 2024-12-09

**Authors:** Rita Marques, Maria dos Anjos Dixe, Patrícia Pontífice Sousa

**Affiliations:** 1 Portuguese Red Cross Health School (Lisbon), Center for Innovative Care and Health Technology, CiTeChare, 1649-023 Lisboa, Portugal; 2 School of Health Sciences, Instituto Politécnico de Leiria, 2411-901 Leiria, Portugal; maria.dixe@ipleiria.pt; 3 Faculty of Health Sciences and Nursing, Universidade Católica Portuguesa, 1649-023 Lisboa, Portugal; patriciaps@ucp.pt

**Keywords:** interventions, comfort, elderly, hospital, nursing

## Abstract

**Background:** The comfort of the elderly in hospital settings requires special attention from the health care professionals involved, particularly nurses, since hospitalization often generates suffering and discomfort. In such contexts, it is essential to consider the specific characteristics of the elderly, taking into account their life experiences and their needs, to promote the health, well-being, and comfort of this population. Hence, the present work aimed to explore the nursing interventions that promote comfort among the elderly in hospital settings. **Methods:** A mixed descriptive exploratory study was conducted through the application of a questionnaire, using intentional non-probabilistic sampling. The study encompassed 55 elderly individuals hospitalized in the medical service of a public hospital located in Lisbon. **Results:** The results show that the participants perceived a reasonable level of comfort (5.65 ± 6.46). The following categories emerged from the content analysis: (1) physical interventions; (2) psycho-spiritual interventions; (3) socio-cultural interventions; and (4) environmental interventions. **Conclusions:** These findings help to understand comfort-promoting nursing interventions in the studied population. It was concluded that, to improve care quality, comforting interventions should focus on the elderly patient’s individuality, through support activities, empowerment, and the preservation/correction of the surrounding environment. Additionally, the provided care should be based on the real needs, expectations, preferences, and values of the elderly individual.

## 1. Introduction

Population aging is a worldwide reality. This phenomenon is caused by an increased average life expectancy, which derives from medical progress and significant improvements in living conditions (quality of housing, sanitary and hygiene conditions, information on healthy lifestyles, diet, and access to health care) [1]. At the same time, different ways of preventing diseases/complications have also emerged, including preventive therapeutic vaccines, complementary diagnostic means, and rehabilitation therapies. These circumstances allow individuals to survive longer and reach more advanced ages, thus enhancing the development of chronic diseases (e.g., osteoarticular, cardiovascular, neurological, respiratory, renal, and neoplastic) [2].

It is estimated that, by 2050, one in six people will be 65 years or older, and part of the elderly population may develop a chronic disease condition [3]. This trend has considerable implications for various spheres of society, particularly for the social, economic, and health domains, given that age is the main risk factor for disabling conditions (e.g., cancer, cardiovascular diseases, neurodegenerative diseases, and osteoarticular diseases) [3]. Most people over the age of 65 have at least one chronic condition, and often two or more [4].

Therefore, demographic aging and the consequent increase in the prevalence of chronic disease are major challenges for the development of health policies [5]. In the 21st century, societies in general, and nurses in particular, should focus on promoting a healthy aging process, based on dignity, comfort, and quality of life [3]. Hence, it is vital to look at aging with a more preventive view, to promote health, autonomy, comfort, and quality of life, especially for those who are hospitalized and facing an important transitional phase in their lives.

Comfort is perceived as a central concept in nursing (both in research and in clinical practice), as well as being the desirable outcome of care provision. Nevertheless, its conceptualization is not yet consensual, since it is a complex concept that varies according to the individuals, contexts, and relationships in question [6]. As it refers to a situational and circumstantial context, its meaning is immediate and dynamic, deriving from several aspects: the experienced circumstances, intrinsic factors that are present in the relationship with oneself, and the individual’s interactions with others/the environment/society [7,8]. On the other hand, experiencing comfort is a positive phenomenon that goes beyond the relief of discomfort—it is an immediate state, which is felt in different domains: physical, environmental, social, and psycho-spiritual [9,10].

In 2018, Veludo defined the concept of comfort as a sensation by reviewing the available literature (108 articles) and performing a hermeneutical data analysis. Regarding the concept’s central components, the following aspects were identified: antecedents—any experience that an individual may undergo, as a result of physical, psycho-spiritual, socio-cultural, or environmental interactions; attributes—security, control, realization of oneself, belonging, peace and plenitude, relaxation, and normality of life; consequences—it strengthens the individuals (increasing their ability to deal with life’s adversities), allows a peaceful death, and improves institutional results [9,11].

In the experience of the hospitalized elderly individual, the process of comforting care is based on a multi-systemic and multi-factorial interaction between the elements involved. This interaction is influenced by the care context, as it is connected with the manners/means used for comforting, as well as each element’s conceptions of comfort/non-comfort. In the interaction between the nurse, the elderly patient, and their family, the nurse’s integrative and intentional action is decisive in meeting the elderly patient’s comfort needs. The comfort needs perceived by the elderly are related to changes in the health/disease process, attitudes towards “oneself and life”, the service’s structure/functioning, and family/significant people [12]. Hospitalization and the confrontation with illness compel the elderly to restructure their reference system and reshape their attitude towards life. As a privileged comfort actor, the nurse’s comforting intervention must be based on respect, considering the Other’s singularity and needs [12,13].

Comforting care is defined as a social, multi-contextual, integrative, individualized, and subjective process, which encompasses multiple dynamic variables, following a logic of commitment, intentionality, mutuality, and continuity. It employs a comprehensive model to accompany the elderly patient, considering the entirety of the caregiver and the entirety of the care recipient. As previously mentioned, because it is based on an encounter/interaction between the involved actors, it is influenced by the care context, where two significant cultural domains emerge, which relate not only to the manners/means used for comforting, but also to the conceptions of comfort/non-comfort [12]. When interacting with the elderly individual and their family, the nurse must consider the patient’s dependence, fragility, and increased vulnerability. Hence, the nurse must acknowledge all the existing socio-affective changes and implications to successfully carry out the proposed nursing interventions.

The process of comforting care stems from the context’s specific environment combined with the characteristics and actions constructed by the participating actors [8]. According to Kolcaba, holistic comfort comprises two dimensions: one consisting of the three states of need satisfaction (relief, tranquility, and transcendence) and the other of the four contexts in which comfort occurs (physical, environmental, socio-cultural, and psycho-spiritual). In this sense, Kolcaba stresses that comfort-promoting interventions should be considered a good practice in nursing care only when the intervention in question is perceived as comforting by the targeted individual, family, or community [9]. Due to the relevance of this phenomenon, several authors consider it to be important for the discipline of nursing, but only Kolcaba has conceptualized it in a middle-range theory. However, although the study is not based solely on this theory, the results of the content analysis make sense in the dimensions of this theory.

Considering the above reality, we sought to answer the following questions: What level of comfort do the elderly feel in a hospital environment? What nursing interventions promote comfort of the elderly in a hospital setting?

## 2. Materials and Methods

### 2.1. Aim and Study Design

The aim is to understand the level of comfort of hospitalized older people and the nursing interventions that promote older people’s comfort in hospital. To this end, we conducted a mixed descriptive exploratory study. To understand the level of comfort of hospitalized elderly people, we opted for a single question on a Likert scale from 0 to 10, where 0 represented no comfort and 10 represented total comfort.

The collected qualitative data were subjected to a content analysis, performed according to Bardin’s recommendations [14]. The analysis process followed the three main steps described by Bardin: Pre-analysis, Exploration of the Material, and Treatment of the Results. In the 1st stage, the content was read several times for the researcher to become familiar with the data. The transcripts were organized and divided into segments that seemed most relevant to the objective of the study. The analysis was performed based on data coding, where significant excerpts were grouped into categories. The categories emerged both deductively, from the theoretical framework, using the dimensions already defined by Kolcaba, namely, physical, psycho-spiritual, socio-cultural, and environmental [9], and inductively, during the reading of the data. The interpretation of the results was based on the research question and framed the nursing interventions in the four dimensions defined by Kolcaba.

This study followed the guidelines of the Mixed Methods Appraisal Tool (MMAT), version 2018 [15].

### 2.2. Participants

This study’s target population consisted of individuals with 65 years of age or more, who had been admitted to the medical service of a public hospital in the Lisbon area, which allowed easy access to the population in a safe and controlled environment.

We opted for an intentional non-probability sampling technique, based on a conscious choice to include/exclude the elements according to their characteristics [16]. As such, the following inclusion criteria were established: elderly (aged 65 or over); suffering from a chronic illness (self-reporting/mentioned in clinical registries); ability to speak/understand Portuguese; cognitive ability for self-assessment (Mini Mental State Examination: >5 points for illiterate patients, >22 points for patients with up to 11 years of schooling, and >27 points for patients with more than 11 years of schooling); hospitalized for more than 24 h (in order to have a better perception of the comforting interventions in the studied context); freely consenting to participate in the study. The individuals were selected by the researcher, who always verified their willingness to participate, through an initial introductory dialog. All the individuals approached who met the inclusion criteria agreed to take part in the study.

The sample’s final size and composition were determined by data saturation, during the analysis process, taking into consideration the richness of the individual experience and the attainment of information redundancy without adding new data [17].

### 2.3. Data Collection

Based on the defined objectives, we designed a questionnaire, which included the sample’s socio-demographic variables (gender, age, marital status, educational/academic qualifications, profession/occupation, and residence/current permanence), as well as clinical variables (history of chronic illness). It also comprised two questions: the first was closed and appraised the participant’s comfort level: “On a scale from 0 to 10, indicate your level of comfort, where 0 is no comfort at all and 10 is the maximum level of comfort”; the second part asked participants an open question: “What gives or could give you more comfort right now?”.

To ensure that the questions were understandable, and to ascertain the answers’ average length, the questionnaire was subjected to a pre-test, accompanied by verbal reflection, conducted on individuals of the target population who did not participate in the study. This process made it possible to identify and correct any ambiguities or difficulties in interpreting the questions, ensuring that the tool was suitable for data collection and easily understood by the participants.

Participants were identified by the nursing team and selected by the researcher according to the inclusion criteria, who always checked their availability to participate, through an initial introductory dialog. All individuals contacted agreed to participate in the study.

The instructions for completing the questionnaire were given by the researcher, and the questionnaire was self-completed in her presence. The answer was free, with no time or space limit. Participants completed the questionnaire after 24 h of hospitalization. Completing the questionnaire took an average of 8 min and data saturation occurred around 5 months after data collection began.

### 2.4. Ethical Statement

As required, the study was approved by the institutional ethics committee of the Universidade Católica Portuguesa (Approval no. 91/2020). All the ethical principles defined in the international Helsinki convention were safeguarded, ensuring respect for each individual and their self-determination. Before carrying out the survey, the researcher provided sufficient information to the participants regarding the study’s purpose, the intended use for the collected data, and data protection. Free consent was obtained from all participants, by means of a consent form.

To maintain impartiality and fidelity in the coding of the data, it was carried out in the presence of two researchers. When in doubt, the opinion of the third researcher was sought.

### 2.5. Statistical Analysis

The qualitative analysis of the data was carried out with the support of the NVivo 15 software, which was used for the organization and management of the qualitative data. NVivo facilitated the process of storing interview transcripts, as well as coding the data and creating categories.

The quantitative data were processed using version 26.0 of the Statistical Package for the Social Sciences (SPSS) software for Windows. A descriptive analysis was carried out and the Mann–Whitney U test and Spearman’s correlation were used to check for differences in the perception of comfort according to gender and age.

## 3. Results

### 3.1. Socio-Demographic Characteristics and Comfort Level

The final sample consisted of 55 hospitalized elderly individuals, with an average age of 70.95 ± 6.46 years (median = 68), and mostly men (31; 56.4%). The majority of the participants were married (39; 70.9%). In terms of educational/academic qualifications, most had basic schooling (40; 74.6%), while only 6 (12.7%) possessed a higher education. All the participants suffered from a chronic illness, with respiratory (13; 23.6%) and cardiovascular (12; 21.8%) diseases prevailing, followed by kidney and neoplastic diseases (both with 7; 12.7%). More detailed information can be found in Table 1.

As regards comfort, on a Likert scale from 0 (absence of comfort) to 10 (maximum comfort), the participants self-perceived a reasonable level of comfort (5.65 ± 6.46), which requires some analysis of its meaning.

Using the Mann–Whitney U test and Spearman’s correlation, we found that the perception of comfort level is not related to gender (U = 352.500; *p* > 0.05), but it increases with age (rs = 0.359; *p* < 0.05).

### 3.2. Findings from the Thematic Analysis

**Figure 1:**
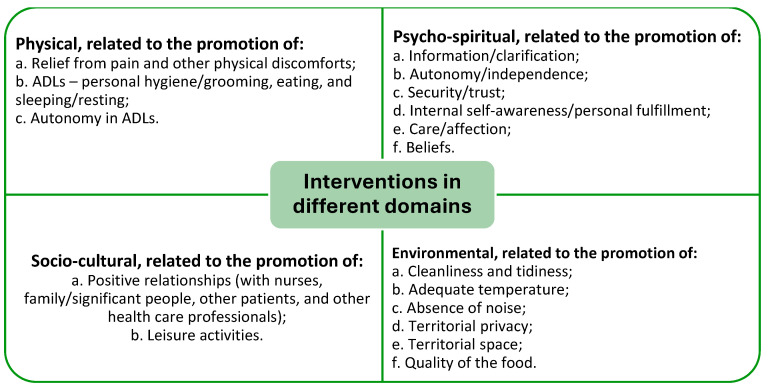
Thematic map showing an overview of the findings.

The transcribed accounts of the elderly individuals are identified by the letter “I” followed by the respective participation number. Through the processing of the collected information, the participants’ discourse was disassembled, organized, systematized, and analyzed to select, group, simplify, and transform the gathered data. From this procedure, four thematic categories emerged, which allow for a better understanding of the nursing interventions that promote comfort among the elderly in hospital settings: (1) physical interventions; (2) psycho-spiritual interventions; (3) socio-cultural interventions; and (4) environmental interventions. An overview of the categories/themes and subcategories/subthemes is shown in Figure 1.

#### 3.2.1. Physical Interventions

There are several interventions in the physical domain that contribute to the comfort of the elderly individual in a hospital setting. Such interventions are related to the promotion of the following aspects:

- Relief from pain and other physical discomforts

Within the physical sphere, various findings were included in the subcategory “relief from pain and other physical discomforts”: not feeling pain (I3; I7; I21; I26; I31; I44; I48; I53); being physically well (I1; I36); taking medication (I24; I49); the nurse’s massage (I37; I43); rehabilitation/physiotherapy (I18; I35; I41); not feeling nauseous (I51).

- Activities of daily living (ADLs)

This subcategory encompassed different elements associated with personal hygiene/grooming [having a clean and tidy body (I2; I16; I33); a hot shower (I8; I44)], eating [being able to eat (I8; I23; I51)], and sleeping/resting [sleeping well (I6; I16; I37; I42); resting/lying down (I31); sitting in an armchair resting (I2)].

- Autonomy in ADLs

There are some findings that refer specifically to the individual’s autonomy in performing ADLs: being able to walk (I1; I34; I52); being able to go to the toilet on my own (I23); being able to carry out everyday activities (I9; I24; I36).

#### 3.2.2. Psycho-Spiritual Interventions

Given the complexity of the elderly individual’s comfort in a hospital setting, many interventions of a psycho-spiritual nature stand out. Such interventions are related to the promotion of the following aspects:

- Information/clarification

In the psycho-spiritual domain, some results fell under the subcategory “information/clarification”: having more information about my problem/situation (I1; I11; I27; I35; I49; I52); being told the truth (I4; I21; I55); knowing that the others are well (I19); being told everything (I33); knowing that my family is well (I30).

- Autonomy/independence

The importance of autonomy/independence-promoting interventions is reflected in the following statements: being independent (I1; I25; I31; I42); being autonomous (I7; I11; I27); being able to carry out my activities on my own (I32); being able to take care of myself (I16; I28).

- Security/trust

The need for interventions that focus on elements associated with security/trust is evident in the following reports: not worrying about anything (I2; I15; I22); knowing that I’m safe (I10; I23); having good medical care (I18; I45); believing that I’m going to make it (I29).

- Internal self-awareness/personal fulfillment

The pertinence of promoting internal self-awareness/personal fulfillment is noticeable in the following accounts: having willpower (I5; I9; I53); will to live (I14); helping others (I27; I46); feeling that I have done everything I could (I29).

- Care/affection

The relevance of performing interventions that promote care/affection is apparent in the following record units: being surrounded by care (I3); the nurses’ affection (I17; I47); the family’s affection (I33; I39); other people’s friendship (I13; I44; I48); the love of my husband (I43); the family’s presence/support (I9; I29); having people who comprehend me (I21; I55); knowing that they care about me (I22); having other people’s attention (I37; I42); having people who understand me (I25); having human people taking care of me (I49; I54); being treated with respect (I24).

- Beliefs

The significance of facilitating beliefs is manifest in the following statements: praying the rosary (I4; I38); praying (I12; I20; I41; I45); listening to Mass (I25); believing in something (I35; I41); believing in God (I40).

#### 3.2.3. Socio-Cultural Interventions

From the individuals’ narratives, it was possible to discern that interventions at a socio-cultural level were essential for their comfort. Such interventions are related to the promotion of the following aspects:

- Positive relationships

The subcategory “positive relationships” includes statements associated with nurses [talking with the nurse (I1; I6); the nurses’ presence (I7; I32)], family/significant people [talking with family members (I2; I8; I19; I51); having visitors (I4; I9; I17; I20; I21; I33; I41; I43); having support from family and friends (I11; I33); the family’s presence (I23; I31; I39; I40; I44); the children’s presence (I14; I22; I23; I47; I52); the grandchildren’s presence (I38); receiving messages/calls via WhatsApp/Facebook from family and friends (I12; I24; I33)], other patients [talking with the other patients (I11; I28; I34; I45)], and other health care professionals [the assistants’ friendliness (I13); regard for my likings/preferences (I25); having thoughtful people/professionals who help me (I35; I46); having competent health care professionals taking care of me (I47; I53)].

- Leisure activities

Several reports mentioned leisure activities as comfort-promoting elements: listening to music (I13; I15; I19; I37); watching television (I14; I26; I43; I54); reading (I16; I36; I50); reading the newspaper (I49).

#### 3.2.4. Environmental Interventions

In the context under study, the individuals involved considered that the environment influences their comfort, as it is a key domain within the sphere of the nurse’s action. As such, environmental interventions aim to promote the following aspects:

- Cleanliness and tidiness

The importance of promoting cleanliness and tidiness is manifest in the following statements: cleanliness (I3); the room being tidy (I34; I36; I38; I41); having a clean bed with washed linen (I42); the environment being organized (I48).

- Adequate temperature

The temperature’s adequacy is also noteworthy and was mentioned in some reports [room with an adequate temperature (I17; I40; I55)].

- Absence of noise

The significance of interventions that facilitate the absence of noise is perceptible in the following accounts: the environment being silent (I4; I16; I28); quiet (I23; I47; I54); calm (I27; I35).

- Territorial privacy

The promotion of territorial privacy was valued by the individuals involved and is present in the following statements: having my own space (I2); having privacy (I15; I26); having privacy in my room (I32); having my own bathroom (I31).

- Territorial space

The elderly also appreciated interventions associated with the notion of territorial space [seeing the sun (I5; I16; I30); seeing the street (I24); looking out of the window at the street (I29; I43)].

- Quality of the food

The quality of the food provided was referred to in some statements made by the participants: having good food/good meals (I18; I28; I39); tasty food (I21; I44); food that looks good (I33); healthy food (I41).

The qualitative findings of this study allowed us to explore factors that may be associated with the participants’ self-reported comfort. Higher levels of perceived comfort may be associated with (i) physical interventions—the absence of pain and other physical discomfort and autonomy in daily activities; (ii) psycho-spiritual interventions—care/affection; (iii) socio-cultural interventions—positive relationships; and (iv) environmental interventions—cleanliness and tidiness. On the other hand, the lower levels may reflect the absence of elements such as (i) physical interventions—performing activities of daily living; (ii) psycho-spiritual interventions—information/education; (iii) socio-cultural interventions—leisure activities; and (iv) environmental interventions—territorial privacy. These results emphasize the importance of targeted, individualized nursing interventions to promote comfort, specifically targeting these factors.

## 4. Discussion

The aim of this study was to understand the level of comfort of hospitalized elderly people and the nursing interventions that promote this comfort in the hospital environment. The final sample consisted of 55 hospitalized elderly individuals, with an average age of 70.95 years, and the majority of whom were men (56.4%). In terms of health conditions, all participants suffered from chronic diseases, particularly respiratory and cardiovascular diseases. In terms of comfort, the participants rated their level of comfort as moderate, with an average score of 5.65 on the Likert scale of 0 to 10, indicating the need for nursing interventions to improve this aspect. Statistical analysis showed that comfort was not related to gender but increased with age.

In the context under study, according to the logic of person-centered care, comfort involves each elderly person identifying in their uniqueness the different ways/means of comforting, which translates into answering the following question: “Right now, what gives you comfort?”. This enables the discovery of possible nursing interventions that promote comfort for the target population. In this logic, the elderly person is seen as a care partner [18].

Our findings systematize the comfort experience in the context of hospitalization, considering the transition lived by the elderly in such settings. Once we had extracted the meaning units from the statements, we grouped those corresponding to each category/subcategory according to their semantic value. The participants identified various manners/means of comforting that, acting simultaneously, gave rise to the feeling of comfort experienced at that moment. The reported manners/means were related to (1) physical, (2) psycho-spiritual, (3) socio-cultural, and (4) environmental interventions, which fit into the four contexts of Kolcaba’s theory [9]. It is evident that these four antecedents of comfort—which originate within the individual or result from the intervention of others—constitute the source of the sensation [11]. The results about nursing interventions that promote comfort in the elderly support the conceptual framework of Kolkalba’s theory, which highlights the need for nurses to structure their care practice around his theoretical constructs. However, further research is recommended to compare and test specific interventions based on this theory.

By analyzing the obtained results, we found that many of the statements about the manners/means of comforting were worded negatively, showing that the absence of certain elements promotes comfort. Nonetheless, the available literature is unanimous in considering that the concept of comfort can be associated with a state of relief/encouragement, rather than the absence of any discomfort, thus acquiring a positive connotation [9,19].

Physical interventions play a crucial role in the comfort of hospitalized elderly people. The analysis revealed three main areas of intervention in the physical domain: relief from pain and other physical discomforts, activities of daily living (ADLs), and autonomy in ADLs. In this sense, comfort is viewed as the result of an intentional action, centered on the control/absence of pain and other physical discomforts, which are thus considered synonymous [20]. Nursing interventions should focus on recognizing the individuality of the suffering experience. Therefore, the nurse’s actions should be guided by the possibility of helping the patient to achieve a state of relief/absence of pain, while also promoting the patient’s autonomy in satisfying their basic human needs [8].

Psychospiritual interventions were identified as essential for promoting comfort among hospitalized elderly people, addressing various aspects that go beyond physical care but are also crucial for patients’ emotional and psychological well-being. The main areas of intervention identified were: information/clarification, autonomy/independence, security/trust, internal self-awareness/personal fulfillment, care/affection, and beliefs.

Hospitalization generates feelings of uncertainty in the elderly and their families, triggering high levels of anxiety and concern. It is an unpredictable situation, which causes insecurity and fear [21]. Such circumstances usually have a strong impact on the psycho-spiritual domain, requiring a process of adaptation. They are a source of suffering, deeply marked by emotional instability [13].

The communication process is involved in the construction of comforting interventions, as a key factor in the interaction. It allows the development of a therapeutic relationship with the patients and their families, which promotes the situation’s understanding. Providing information and clarification regarding the patient’s clinical status, in a rigorous and up-to-date manner, allows the patient to have more control over the provided care and facilitates their adaptation to the experienced circumstances [13,22]. While constructing a comforting intervention, it is important to include pertinent information, according to the needs and concerns expressed by the patient, since a lack of knowledge generates insecurity and uncertainty [13]. Information is the basis for the patient’s autonomous decisions, allowing the individual to consent to, or refuse, the proposed health measures/procedures [10]. With respect to comforting care, verbal and non-verbal communication is a fundamental tool, which requires nurses to be empathetic and close to their patients, interact with them, and establish a partnership [23].

The importance of promoting autonomy/independence is widely acknowledged, since it has a major influence on the individuals’ dignity, integrity, and freedom, as well as being a central component of their general well-being [24]. In hospitalization settings, the individual may become more fragile, and there exists a clear need for care that seeks to maintain autonomy and stimulate the individual’s abilities. Accordingly, the patient should actively participate in the care provision process, in as independent a manner as possible [13].

Still in the psycho-spiritual domain, Kolcaba recognizes the significance of internal self-awareness/personal fulfillment, which encompasses self-esteem, the meaning of life, sexuality, the concept of oneself, and the relationship with a higher being [9]. Given that the inner strength of each individual is crucial for a positive day-to-day experience, its promotion is essential to safeguard human dignity [9].

The elderly value displays of care and affection from nurses/significant people (e.g., tenderness, friendship, love, understanding, concern, attention, humanism, and respect). For the elderly, relational attitudes associated with care and affection are human qualities that promote comfort [8]. This reinforces how nursing care is built within the context of an encounter between the individual and the nurse/significant person.

Spiritual interventions make sense in terms of promoting beliefs and values. They emerge in an attempt to facilitate a balanced and meaningful life [13]. Each individual, when confronted with their own existence, more specifically with disease and hospitalization, adopts a kind of spirituality, or a particular way of being, which allows them to cope better with problems [25]. The expression of spirituality is related to the individuality of each elderly person. It provides an inner strength that gives meaning and significance to life [13].

Socio-cultural interventions play a crucial role in the comfort of hospitalized elderly people, as revealed by the participants’ narratives. These interventions address aspects related to social interaction, affective support, and activities that promote emotional and psychological well-being. The main categories that emerged were positive relationships and leisure activities.

In the socio-cultural domain, interpersonal, family, and social relationships have a positive influence and generate comfort [9]. Comforting relationships can come from a variety of sources (e.g., nurses, doctors, other health care professionals, family members, and significant people), depending on the individual’s needs or the circumstances of the moment [26]. In this sense, in an intentional search for the uniqueness/particularity of each elderly individual, the following main predictors of comfort emerge: availability, trust, provided information, acknowledgment as a person, closeness, presence, showing interest, sympathy, and the implementation of non-routine interventions [26,27]. Maintaining positive relationships with family and friends makes the individuals feel cared for, loved, esteemed, valued, and supported, giving them a sense of belonging [8].

Establishing an authentic relationship that promotes the situation’s understanding allows for a humanized aid, through an appropriate response, adapted to the experienced circumstances. This help can be mobilized by nurses, using the social support network, offering emotional support, and providing information to family members [23].

Filling the period of hospitalization with leisure activities, which facilitate distraction/recreation, diminishes the patient’s suffering [28], and promotes psycho-spiritual and socio-cultural comfort [12]. In this context, leisure has several purposes: distraction, rest, stress reduction, energy renewal, and recreation [28]. The present study highlights the following comfort-promoting activities: listening to music, watching television, and reading. These activities seem to minimize the undesirable effects of hospitalization, thus being beneficial to health. They contribute to the reduction in pain and anxiety, while increasing the individuals’ well-being and comfort [10,29].

Environmental interventions play a significant role in the comfort of hospitalized elderly people, standing out as one of the main domains within nursing practice. Participants’ perceptions indicate that the physical environment in which the elderly are placed has a direct impact on their well-being and comfort. Nursing interventions can be directed towards promoting the following environmental conditions: cleanliness and tidiness, adequate temperature, the absence of noise, territorial privacy, territorial space, and the quality of food.

Focusing on organizational, structural, and operational conditions, environmental interventions are related to the humanization of the hospital’s physical environment. As such, the following aspects are particularly significant in the environmental domain: cleanliness and tidiness (of the bed and room), adequate temperature, the absence of noise, privacy (in terms of space, room, and bathroom), territorial space (e.g., access to a view from the room), and the quality of food (considering taste, appearance, and nutritional value).

Structural and organizational deficiencies, particularly those associated with environmental conditions such as light, noise, equipment/furniture, color, temperature, and natural/artificial elements, have been identified in other studies as factors that limit comfort and may fall outside of the nurse’s direct control [9,12,19]. However, these deficiencies do not hinder the construction of comforting actions, as nursing interventions can still humanize care and address the elderly’s needs [12]. By focusing on aspects within their control, nurses can create a more comfortable and supportive environment, even in less-than-ideal physical settings.

The results obtained provide a comprehensive overview of the different dimensions of comfort and the nursing practices that can be improved to enhance the experience of elderly patients in hospitals. However, to adequately measure the level of comfort, nurses must use validated assessment scales in their nursing practice, adapted to the specific characteristics of the population.

## 5. Conclusions

Comfort is a desirable state, influenced by the circumstances and moment experienced. In this study, participants reported a reasonable level of overall comfort, with key factors identified across four domains: physical, psycho-spiritual, socio-cultural, and environmental. These domains reveal the complex nature of comfort, highlighting the multiple factors that nursing interventions can target to enhance well-being.

Nursing interventions should address these four domains, focusing on high-quality care that respects each patient’s individuality. Interventions should support, empower, and protect the elderly patient by considering their specific needs, preferences, and values. By taking a holistic approach, nurses can improve both the physical and emotional well-being of hospitalized elderly individuals.

These findings emphasize the importance of individualized interventions that address all comfort-related domains. It is crucial for nursing practices to be comprehensive and responsive to the diverse needs of elderly patients, ensuring that care promotes both physical and emotional comfort. Further research, involving larger samples and different methodologies such as observation and interviews, is needed to deepen our understanding of comfort-promoting interventions. However, future studies should use an instrument to measure the different dimensions of comfort that has been validated for the population to statistically explore the relationship between the qualitative and quantitative results. Additionally, exploring nurses’ perspectives on comfort interventions for elderly patients with chronic conditions would be valuable.

In conclusion, this study underscores the need for nursing practice to adapt to the multifaceted comfort needs of elderly patients. By tailoring interventions to address physical, emotional, and environmental aspects, nurses can significantly improve the quality of care, ensuring that elderly patients experience a more comfortable and supportive hospital stay.

## Figures and Tables

**Table 1:** Socio-demographic and clinical characteristics of the sample (n = 55).

Variables		n (%)
Sex	Male	31 (56.4%)
	Female	24 (43.6%)
Marital status	Single	6 (10.9%)
	Married	39 (70.9%)
	Divorced/separated	1 (1.8%)
	Widowed	9 (16.4%)
Educational qualifications	No schooling	1 (1.8%)
	1st cycle (4th year)	19 (34.3%)
	2nd cycle (6th year)	10 (18.2%)
	3rd cycle (9th grade)	12 (21.8%)
	Secondary (12th grade)	6 (10.9%)
	Higher education	7 (12.7%)
Chronic illness	Cardiovascular disease	12 (21.8%)
	Metabolic disease	3 (5.5%)
	Respiratory disease	13 (23.6%)
	Renal disease	7 (12.7%)
	Cerebrovascular disease	6 (10.9%)
	Neurological disease	3 (5.5%)
	Liver disease	1 (1.8%)
	Neoplastic disease	7 (12.7%)
	More than one chronic disease	2 (3.6%)

## Data Availability

This work is part of the first author’s post-doctoral project. All data generated/analyzed during the study were incorporated into the article.
